# Effects of Antidepressants on Sleep

**DOI:** 10.1007/s11920-017-0816-4

**Published:** 2017-08-09

**Authors:** Adam Wichniak, Aleksandra Wierzbicka, Małgorzata Walęcka, Wojciech Jernajczyk

**Affiliations:** 1 Third Department of Psychiatry, Institute of Psychiatry and Neurology, Sobieskiego 9, 02-957 Warsaw, Poland; 2 Center for Sleep Medicine, Department of Clinical Neurophysiology, Institute of Psychiatry and Neurology, Sobieskiego 9, 02-957 Warsaw, Poland

**Keywords:** Depression, Sleep, Antidepressants, Insomnia

## Abstract

**Purpose of Review:**

The aim of this review article was to summarize recent publications on effects of antidepressants on sleep and to show that these effects not only depend on the kind of antidepressant drugs but are also related to the dose, the time of drug administration, and the duration of the treatment.

**Recent Findings:**

Complaints of disrupted sleep are very common in patients suffering from depression, and they are listed among diagnostic criteria for this disorder. Moreover, midnocturnal insomnia is the most frequent residual symptom of depression. Thus, all antidepressants should normalize sleep. However, at least in short-term treatment, many antidepressants with so-called activating effects (e.g. fluoxetine, venlafaxine) may disrupt sleep, while others with sedative properties (e.g., doxepin, mirtazapine, trazodone) rapidly improve sleep, but may cause problems in long-term treatment due to oversedation.For sleep-promoting action, the best effects can frequently be achieved with a very low dose, administered early enough before bedtime and importantly, always as a part of more complex interventions based on the cognitive-behavioral protocol to treat insomnia (CBT-I).

**Summary:**

For successful treatment of depression, it is necessary to understand the effects of antidepressants on sleep. Each physician should also be aware that some antidepressants may worsen or induce primary sleep disorders like restless legs syndrome, sleep bruxism, REM sleep behavior disorder, nightmares, and sleep apnea, which may result from an antidepressant-induced weight gain.

## Introduction

Depression is a severe and common mental disorder with 12-month prevalence as high as 3.2% in subjects without comorbid physical disease and 9.3 to 23.0% in subjects with chronic medical conditions [1]. Despite its frequent occurrence, high likelihood of a chronic course, negative impact on quality of life and ability to work, and strong association with an increased suicide risk, the available treatment options for depression are still not satisfactory for many patients. The most neglected pharmacological needs in the treatment of depression are the lack of early-onset response to the treatment, the moderate response and low remission rate to the first antidepressant trial, and side effects which frequently cause treatment non-compliance [2]. Among the most common side effects of antidepressants and residual symptoms leading to incomplete remission from depression are those related to sleep. The aim of this review article is to summarize the literature published in recent years on how antidepressants affect sleep, as an addition to our [3] and previous reviews on this topic [e.g. 4–8]. We also summarize recent data which has shaped our personal view on the use of antidepressants in treating insomnia in depressed and non-depressed subjects.

## Polysomnographic Sleep Studies in Depression

The most detailed information on sleep in depression was provided by studies using polysomnography (PSG), that is considered the gold standard for sleep assessment. Based on registration of the three physiological parameters such as the brain (EEG), muscle (EMG) and eye movements (EOG) bioelectric activity, PSG allows human sleep to be scored into sleep stages. Subsequently, it is possible to calculate several sleep parameters (Table 1) that express sleep continuity, sleep depth, and distribution of sleep stages.

**Table 1:** Definitions of sleep parameters based on scoring of sleep stages in polysomnographic recording, and used to describe the sleep architecture

Parameters of sleep continuity	
Sleep latency	Time from start of the recording (“lights out”) to the onset of sleep. Normal values are typically below 30 min in young and below 45 min in elderly patients.
Total sleep time (TST)	The total time spent asleep during the sleep episode. This is equal to the time in bed less the awake time. In insomnia research as shortened sleep time are considered usually values below 6.5 h in young and below 6 h in elderly patients (these values are not applicable to short sleepers)
Sleep efficiency (SE)	The ratio of total sleep time to time in bed expressed as a percentage of time spent asleep during the recording period. Normal values are typically above 90% in young and above 85% in elderly patients.
Wake after sleep onset (WASO)	The total time scored as awake occurring after the sleep onset. Typically WASO should not exceed 30 min.
Parameters of sleep depth	
Total and relative amounts of stage N3	Total duration in minutes and as percentage relative to total sleep time of sleep stage N3. The amount of stage N3 decreases with older age, normal values are around 10% for elderly and 20–25% for young subjects.
Delta sleep ratio	The ratio of slow wave sleep in the first and second sleep cycle. Normally, values exceed 1.1
Parameters of REM sleep	
REM latency	The number of minutes from the onset of sleep to the onset of the first REM sleep period. Reduced values are typically below 65 min in young and 50 min. in elderly patients.
Total and relative amounts of stage REM	Total duration in minutes and as percentage relative to total sleep time of sleep stage REM. Normal values are 20–25%.
REM density	The ratio of the intensity of rapid eye movements phasic activity (number and duration of rapid eye movements) to duration of REM sleep, e.g., can be expressed as number of rapid eye movements per minute of REM sleep.

**Fig. 1:**
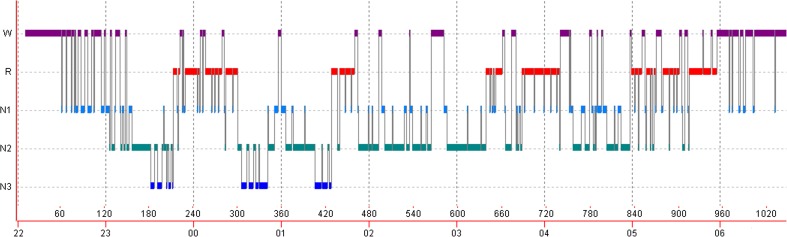
Graph (hypnogram) representing changes of sleep stages in the course of night in a depressed patient. Sleep in depression is characterized by disturbances of sleep continuity (prolonged sleep latency, increased number and duration of awakenings from sleep, early morning awakening), reduction of deep (slow wave sleep), and disinhibition of REM sleep, with shortening of REM latency and prolongation of the first REM period. Y-axis represents sleep stages: *W*- wake, *R*- REM sleep, *N1* – stage 1 NREM sleep, *N2* – stage 2 NREM sleep, *N3* -stage 3 NREM sleep (slow wave sleep, deep sleep), and *X-axis* represents time and pages of sleep recording

Graphically, the sleep architecture is displayed with a graph that is called hypnogram (Fig. 1).

Patients with depression show abnormalities of sleep parameters across all three groups. Disrupted sleep continuity manifests as prolongation of sleep latency, increased number, and duration of awakenings from sleep expressed as increased wake after sleep onset (WASO) time, decreased sleep efficiency, and early morning awakenings. Early morning, awakening together with altered distribution of REM sleep is considered a biological marker of circadian rhythm disturbances in depression and is a characteristic biological marker of depression with melancholic features [9]. Sleep depth is substantially reduced in depressed patients. Furthermore, the distribution of deep sleep scored in PSG as sleep stage N3, also called delta or slow wave sleep (SWS), is altered in depressed patients. In healthy subjects, the highest delta wave activity in EEG can be observed in the first sleep cycle, whereas in depressed patients there is a frequent shift of delta activity from the first to the second sleep cycle. It is expressed in sleep parameters as a reduced delta ratio (ratio between delta wave activity in the first and second sleep cycle). Alterations of REM sleep are the most prominent feature of sleep architecture in depressed subjects. They include shortened REM sleep latency, increased REM sleep time (especially in the first sleep cycle that is usually very short in healthy subjects), and increased REM sleep density. The recent meta-analysis summarizing the current evidence from studies using PSG about sleep architecture in mental disorders has confirmed that disturbed sleep is a core symptom of depression. It was found that affective disorders and major depression were associated with alterations in most variables compared to healthy controls [10••]. Although none of the sleep parameters was specific to depression, the high prevalence and severity of sleep abnormalities in depressed patients are of a great clinical importance. The complaints of insomnia are present in 60–90% of patients with major depression, depending on the episode’s severity. When it comes to bipolar disorder, insomnia is present during a depressive episode in 60% of patients, while 20–30% suffer from prolonged sleep (hypersomnia) and increased daytime sleepiness [11, 12]. Fortunately, in most patients, sleep disturbances diminish with the improvement of depressive symptoms, especially if the clinical improvement is related to the recurrence of interest and pleasure in everyday activities. Because it is usually related to the substantially increased physical daytime activity, it increases homeostatic sleep need, which improves sleep depth and duration. However in many patients, difficulties with sleep persist. The Sequenced Treatment Alternatives to Relieve Depression (STAR*D) study, which included a large sample of outpatients with non-psychotic MDD who responded without remitting after up to 12 weeks treatment with citalopram, found that midnocturnal insomnia was the most commonly observed residual symptom of depression, as it was still present in 79% patients [13].

The observations on the high prevalence of subjective insomnia complaints and objective worsening of sleep architecture in PSG studies in depressed patients are important for the choice of pharmacological treatment. Antidepressant drugs substantially differ in their acute effects on sleep. Some of them alleviate sleep disturbances, but other may disrupt sleep, which is related to poor treatment compliance. Persistent insomnia symptoms may also result in unfavorable clinical outcome, e.g., increased suicide risk [14]. Therefore, it is important to know what is the preferred pharmacological treatment in a depressed patient with clinically relevant insomnia symptoms.

## Effects on Antidepressants on Sleep

It is well known that some classes of antidepressant drugs may deteriorate sleep quality mainly due to activation of serotonergic 5-HT2 receptors and increased noradrenergic and dopaminergic neurotransmission (Table 2). Among them, most prominent are serotonin and norepinephrine reuptake inhibitors (SNRI), norepinephrine reuptake inhibitors (NRI), monoamine oxidase inhibitors (MAOI), selective serotonin reuptake inhibitors (SSRI), and activating tricyclic antidepressants (TCA).

**Table 2:** Effects of antidepressants on sleep

Drug class	Sleep continuity	SWS	REM latency	REM sleep	Mechanism of action related to effect on sleep
Sedative TCA (e.g., amitriptyline, doxepin, trimipramine)	↑	↑	↑	↓	antihistaminergic effect, inhibition of serotonin, and norepinephrine reuptake
Activating TCA (e.g., imipramine, desipramine)	↓	↓	↑	↓	inhibition of serotonin and norepinephrine reuptake
MAOI (e.g., tranylcypromine, moclobemide)	↓/0	?	↑	↓	inhibition of monoamine oxidase enzyme
SSRI (e.g., fluoxetine, escitalopram, paroxetine, sertraline)	↓/0	0/↑	↑	↓	selective inhibition of serotonin reuptake
SNRI and NRI (e.g., venlafaxine, duloxetine, reboxetine)	↓	0//↑	↑	↓	inhibition of serotonin and norepinephrine reuptake
Agomelatine	↑	↑	0	0	agonism at melatonin M1 and M2 receptors, antagonism at serotonergic 5-HT2C receptors
Bupropion	0/↓	0/↑	0/↓	0/↑	inhibition of norepinephrine and dopamine reuptake
Sedative antidepressants (e.g., mirtazapine, trazodone)	↑	↑	0	0	antihistaminergic effect, antagonism at serotonergic 5-HT2A receptors
Vortioxetine	0/↓	?	↑	↓	inhibition of serotonin reuptake and modulation of serotonergic receptors activity

*TCA* tricyclic antidepressants, *MAOI* monoamine oxidase inhibitors, *SSRI* selective serotonin reuptake inhibitors, *SNRI* serotonin norepinephrine reuptake inhibitors, *NRI* norepinephrine reuptake inhibitors, ↑ increase, ↓ decrease, 0 no or minimal effect, ? unknown

On the contrary, antidepressants with antihistaminergic action, like sedating TCA, mirtazapine, mianserine, or strong antagonistic action at serotonergic 5-HT2 receptors, like trazodone and nefazodone quickly improve sleep. Some patients show improvement of sleep quality already after the first drug dose [15], which was specially discussed for mirtazapine as related to the faster onset of antidepressant action [16].

In a recent review article on the prevalence of treatment emergent insomnia and somnolence in depressed patients, it was shown that subjective complaints of insomnia or daytime somnolence were frequent in patients suffering from depression or anxiety disorders treated with SSRI and SNRI [16].

Based on data from the US Food and Drug Administration (FDA) study register [16], the average prevalence of treatment-emergent insomnia in clinical trials with SSRI was 17% as compared to 9% out of patients randomized to the placebo arm. The average rate of treatment emergent somnolence in patients being treated with SSRI amounted to 16% as compared to 8% out of patients receiving placebo. The lowest rate of treatment emergent insomnia complaints (below 2%) was reported in the study with citalopram. The highest rate of treatment-emergent insomnia and somnolence was found in patients suffering from obsessive-compulsive disorder (OCD) being treated with high-dose fluvoxamine, 31 and 27%, respectively [17••]. In clinical trials with SNRI, treatment-emergent insomnia was reported on average in 13% out of SNRI-treated patients as compared to 7% out of the placebo arm and treatment-emergent somnolence in 10% of SNRI-treated patients in comparison to 5% out of patients receiving placebo. Both treatment-emergent insomnia and somnolence were the most frequent (both equal to 24%) in patients with generalized anxiety disorders treated with venlafaxine. The lowest rate of treatment emergent insomnia and somnolence (both below 2%) was reported for levomilnacipran. On the contrary to the treatment with SSRI and SNRI, in clinical studies with sedating antidepressants, e.g., mirtazapine and trazodone, the reported prevalence of treatment-emergent insomnia complaints in patients with major depressive disorder (MDD) was very low (below 2%). However, the rate of treatment-emergent somnolence was very high, 54% in patients being treated with mirtazapine as compared to 18% out of patients in the placebo arm and 46% in patients being treated with trazodone as compared to 19% out of patients receiving placebo. It is important to note that acute effects of antidepressants on sleep are reflected not only in the patients’ subjective complaints but they can also be demonstrated in studies with PSG (Table 2). While SSRI, SNRI, and activating TCA increase REM latency, suppress REM sleep, and may impair sleep continuity, sedating antidepressants decrease sleep latency, improve sleep efficiency, increase SWS, and usually have little or no effect on REM sleep [3, 6, 7, 17••]. Although both the sleep-disrupting and sleep-promoting effects of the antidepressants are the strongest only in the first few weeks of treatment, in some patients they may persist, aggravating insomnia complaints or causing daytime somnolence [18]. Therefore, for the depressed patients with clinically significant insomnia, a treatment with a sedative antidepressant is usually more recommended [19]. It was recently shown that such treatment significantly reduces the need to use benzodiazepines in patients with MDD [20•]. Such an approach, combination treatment with benzodiazepines and SSRI/SNRI is often necessary to reduce anxiety and insomnia as early as in the first week of treatment. However, there is a related risk that the patient suffering from depression and insomnia will not be able to stop such treatment after 14–28 days of therapy and will develop a dependence [21, 22]. On the other hand, because the sleep complaints usually improve after a few weeks of effective treatment of depression with SSRI/SNRI, it is important to consider whether the use of hypnotics is not a better short-term treatment option for a patient than risking oversedation during treatment with a sedative antidepressant. The sedating effect of those antidepressants is usually an increasing problem in long-term maintenance treatment, frequently resulting in a need to reduce the drug dose. It may substantially diminish the efficacy of the maintenance treatment. The sedative antidepressants may also induce a weight gain, what is particularly shown for mirtazapine but not for trazodone [23].

Agomelatine should be considered as an alternative approach to the treatment of depressed patients with marked insomnia symptoms. Agomelatine is a non-sedative antidepressant drug exerting agonistic action at melatonergic M1 and M2 receptors, and antagonistic action at serotonergic 5-HT2c receptors [24]. Such pharmacodynamic profile is related to sleep-promoting action without the risk of sedation and weight gain. In comparison to escitalopram, agomelatine is known to improve sleep latency after both short (after 2 weeks) and long (after 24 weeks) treatment. Moreover, both drugs differ significantly in their effect on sleep continuity. In the second week, agomelatine slightly improves sleep continuity (increased total sleep time and sleep efficiency) and escitalopram worsens it [25]. Moreover, treatment with agomelatine is not related to the suppression of REM sleep: it restores cyclical sleep profile, may increase the amount of SWS, and most importantly leads to the improvement of daytime alertness [26].

Effects on sleep has recently been also reported for a vortioxetine, with clinical action mediated mainly by selective blockade of serotonin reuptake and direct modulation of serotonergic receptors activity (such as 5-HT3, 5-HT7, 5-HT1D, and 5-HT1B) [27]. In a study which has compared the effects of vortioxetine and paroxetine to the placebo in a group of 24 healthy male volunteers, it has been shown that the vortioxetine dose of 20 and 40 mg similarly to the paroxetine dose of 20 mg suppresses REM sleep by increasing REM sleep latency and diminishing duration of REM sleep. Both drugs also decrease total sleep time and increase duration of sleep stage N1. These negative effects of vortioxetine on sleep continuity are significant only for the higher dose [28•]. According to the FDA clinical trial register, the rate of treatment-emergent insomnia complaints or somnolence during the therapy with vortioxetine is lower when compared to SSRI and SNRI drugs [17••].

The use of antidepressants, also those with sedative properties, may impair sleep due to the induction of sleep disorders or worsening already existing ones. Mianserin and mirtazapine may induce restless legs syndrome in as many as 28% of patients [29]. Treatment-emergent RLS has also been described for SSRI and venlafaxine [30]. SSRI, SNRI, and TCA are known to induce or exacerbate sleep bruxism and disturb regulation of muscle tone during REM sleep, causing REM sleep without atonia, which may induce or worsen REM Sleep Behavior Disorder [3, 6]. Moreover, although antidepressants are recommended for the treatment of post-traumatic sleep disorder, they can induce nightmares. We observe this side effect most frequently during the treatment with mirtazapine, just as it was recently reported [31]. Finally, antidepressants inducing weight gain are contraindicated in patients with sleep apnea, that is an overlooked but frequent sleep disorder in people suffering from mental illness [32].

## Treatment of Insomnia Disorder with Low-Dose Antidepressants

Insomnia belongs to the most frequent disorders of the brain [33]. In industrialized countries, approximately 6% of the adults suffer from insomnia as a disorder [34] and as many as 50% may suffer from transient insomnia symptoms [35•]. Although insomnia is not regarded as a severe mental disorder, it shares many features with depression. In order to offer a patient an effective treatment of insomnia, there is a need for a broader perspective, one that reaches far beyond the prescription of hypnotics. Current treatment guidelines [36••] strongly recommend the use of cognitive-behavioral therapy (CBT-I) as the initial treatment for chronic insomnia disorder and only short-term use of the sleep-promoting drugs in patients with insufficient response to CBI-I. However, in daily clinical practice, the use of pharmacotherapy for insomnia is very common. The most frequently used drugs to treat insomnia aside from benzodiazepines and non-benzodiazepine (eszopiclone/zopiclone, zaleplon, zolpidem) hypnotics are sedative antidepressants. However, due to the lack of methodologically sound randomized clinical trials in insomnia, only one of them, doxepin, is approved by FDA for the treatment of sleep maintenance insomnia. Furthermore, recent recommendations discourage the use of other drugs from this class than doxepin for the insomnia treatment [37••]. In our opinion, sedative antidepressants are a valuable treatment option of insomnia in a situation in which despite being in CBT-I therapy, the patient still requires sleep-promoting drugs more than 3–4 times per week. The use of sedative antidepressants should be also considered when there is a comorbid mood or anxiety disorder because such patients are at increased risk of developing hypnotic dependency. Moreover, in many insomnia patients, physiological parameters, e.g., hormone secretion, whole body, and brain metabolic rate, are altered in a similar fashion to the depressed ones what is called a hyperarousal [38, 39], supporting the use of sedative antidepressants to treat such patients. The pros and cons of using sedative antidepressants in insomnia patients were discussed extensively in the earlier papers. This is especially true for trazodone that is very often used as a sleep-promoting drug [3, 39–42]. Frequently expressed concern with the usage of sedative antidepressants in insomnia is that their side effect profile and interactions with other drugs may be underrated [40]. Indeed, although there is evidence for efficacy of sedative antidepressants to promote sleep, for example for TCA in a form of a recent meta-analytic study [43•], it is important to remember that these drugs should be used in insomnia patients only in a very low dose, e.g., for doxepin as low as 3 to 6 mg or 25–50 mg for trazodone. Many psychiatrists are astonished that a sedative antidepressant can promote sleep in such a low dose. Firstly, it should be noted that such low doses are appropriate only for patients with primary insomnia. In the presence of a comorbid mood disorder, the antidepressants have to be used in a recommended therapeutic dose [42]. Secondly, such treatment should be used only when combined with behavioral interventions from CBT-I protocol. When a patient restricts time in bed and uses stimulus control technique, even low-dosage pharmacological treatment starts to work. Thirdly, to be effective in treating sleep-onset insomnia, sedative antidepressants have to be taken much earlier than hypnotics in regard to their pharmacokinetics, especially the time they take to reach the maximum serum concentration (Cmax). It usually means at least 2 hours before sleep (in the case of more rapid drug action the patient should be encouraged to shorten this time). In our opinion, sedative antidepressants are a safe class of drugs when given in low doses. We use them in many patient groups where hypnotics are contraindicated, e.g., in the elderly patients, in patients with sleep apnea and in patients with a history of alcohol and substance abuse. Despite the fact that the use of atypical antipsychotics, mostly quetiapine [44], is increasing for treatment of insomnia accompanying bipolar disorder and schizophrenia, we hold the conviction that sedative antidepressants are a valuable treatment option for such patients as well. Based on our clinical experience and review of published case reports, we believe that the use of sedative antidepressants in a low dose is not related to the increased risk of phase shift in bipolar disorder [45]. Moreover, we have observed that for the treatment of insomnia low doses (5–10 mg) of citalopram administered in the morning can be an alternative to sedative antidepressants with good treatment effects [46].

## Conclusions

Disturbed sleep is a core symptom of depression and its normalization is necessary to achieve remission from the illness. In the long term, all antidepressants which show clinical efficacy improve sleep secondary to improvement of mood and daytime activity. However, in the short term, while some of them may impair sleep due to the activating effects, other may improve sleep due to the sedative properties. Although sleep-promoting action is desired in depressed patients with coexisting anxiety or insomnia, it may be problematic during the maintenance treatment after recovery from depression due to oversedation. Thus, it is necessary to understand the effects of these drugs on the sleep and daytime alertness. It is particularly noteworthy that for sleep-promoting effect, it is sufficient to use a sedative antidepressant in a low dose. In such dose, these drugs can be also combined with other antidepressants as an alternative to hypnotic drugs, especially if there is a clinical necessity to promote sleep for longer than 2–4 weeks with a frequency higher than 3–4 times per week.
